# Combining extraction and cultivation methods for soil seed bank analysis increases number of captured species and their similarity to above-ground vegetation

**DOI:** 10.3389/fpls.2024.1500941

**Published:** 2025-01-20

**Authors:** Petr Plohák, Hana Švehláková, Barbara Stalmachová, Miroslava Goňo, Tomáš Dvorský

**Affiliations:** ^1^ Department of Environmental Engineering, Faculty of Mining and Geology, VSB - Technical University of Ostrava, Ostrava, Czechia; ^2^ Department of Electrical Power Engineering, Faculty of Electrical Engineering and Computer Science, VSB–Technical University of Ostrava, Ostrava, Czechia

**Keywords:** post-mining landscape, above-ground vegetation, soil seed bank, extraction method, cultivation method, similarity of species composition

## Abstract

**Introduction:**

Analysis of aboveground vegetation and soil seed bank is an important source of data on the state and dynamics of vegetation. It is especially important in landscapes exposed to disturbances, which have lost their functions. For our research, a post-mining area in the region of the Upper Silesian Black Coal Basin was selected, whose relief and ecosystems are strongly disturbed by underground mining and are currently also affected by ongoing climate change.

**Method:**

Data collection for our research took place in the territory of two waterlogged subsidence basins in the Karvina region, Czech Republic. We evaluated 30 phytosociological releves using techniques of Zurich – Montpellier school and 540 soil cores using cultivation and extraction method.

**Results:**

In the above-ground vegetation, 115 plant species were identified. By cultivating soil samples, we determined 60 species from 1,487 seedlings, by extraction method 66 species from 5,999 seeds. A statistically significant effect of the presence of the tree layer on the number of species obtained by the extraction method was demonstrated. There is also a statistically significant difference between the selected analysis methods in terms of the length of the captured seeds and their seed mass.

**Discussion:**

The construction of a rarefaction curve demonstrated that the use of cultivation and extraction methods leads to a greater capture of soil seed bank species. The similarity between the species composition of aboveground vegetation and the soil seed bank correspond to similarities observed in other studies from degraded habitats. Very low similarity between the species of the soil seed bank from cultivation and extraction method is probably caused by the highly variable distribution of seeds in the soil in time and space.

## 1 Introduction

The mining of industrial minerals, particularly hard coal, results in extensive and permanent transformations of the landscapes where it occurs. However, these altered territories present a significant challenge for future restoration, not only in terms of ecological and landscape recovery but also from a socio-economic perspective. Landscape restoration efforts are now set against the backdrop of pronounced global climate change, characterized by increasingly extreme weather events such as droughts, floods, torrential rainfall, seasonal moisture deficits, and other adverse conditions. In this context, effective reclamation strategies, incorporating both managed and natural succession, can play a pivotal role. By integrating with the surrounding functional landscape segments that remain undisturbed by mining activities, these strategies can significantly mitigate the negative impacts of such extreme conditions. The soil seed bank holds critical importance for succession processes and the broader landscape restoration effort. Yet, despite its potential, it has received limited attention in current reclamation practices.

In the Ostrava-Karviná District, a mining technology known as controlled collapse was predominantly used for financial and operational reasons. This method results in the formation of subsidence basins on the surface, with their size and depth directly proportional to the intensity and extent of the mining galleries (Stalmachová and Pierzchała, 2011). In some cases, however, the basins can be even more extensive (Havrlant, 2013). In areas with high groundwater levels or within floodplains, these subsidence basins often become waterlogged, leading to the creation of new water bodies of varying sizes and depths (Stalmachová and Pierzchała, 2011). Historically, such basins were frequently rehabilitated - filled with tailings or soil. In some instances, however, changes in local hydrology have transformed these basins into wetland habitats. With careful management, subsidence basins can foster the development of rare habitats and ecosystems, supporting diverse plant and animal species while serving as valuable wetland resources for local communities. However, the long-term ecological outcomes of these areas remain challenging to predict. Continuous monitoring and adaptive management are essential to ensure their ecological sustainability.

The ability of plant species’ seeds to persist in soil in a viable state allows them to endure unfavorable germination conditions, maintain population genetic diversity, and influence the trajectory of secondary succession in habitats. As such, soil seed bank research plays a pivotal role in habitat restoration processes (Bossuyt and Honnay, 2008; Pakeman and Small, 2005). When utilizing soil covers for reclamation, it is crucial to evaluate the soil’s origin to avoid the introduction of undesirable seeds from invasive or expansive species, which could hinder succession. Vegetation development on tailings typically follows a primary succession pathway, with its direction and rate largely determined by the availability of plant propagules in surrounding habitats. Consequently, understanding the composition of above-ground vegetation in reclaimed areas is essential for guiding successful restoration efforts.

Soil seed banks can be analyzed using two main methods: seedling germination and seed extraction. However, each method has its advantages and disadvantages. In addition to seed properties such as length, width, and seed mass, soil and climate factors also affect the results of these methods. According to Gandía et al. (2022), the extraction method captures significantly more seeds in drier conditions, where seeds do not require quick germination and can leverage dormancy, but the use of both methods is suitable for a more accurate assessment of soil seed bank composition. In humid regions with constant soil seed bank recovery, where seeds avoid the dormant state, the differences blur, and both methods are equally suitable for determining soil seed bank composition.

Although seed extraction is effective for larger seeds, it can result in the loss of smaller seeds (Brown, 1992; Mesgaran et al., 2007; Price et al., 2010). Gonzalez and Ghermandi (2012) recommend using this method only for seeds larger than 1 mm. Additionally, not all undamaged seeds are necessarily viable, leading to overestimation of the soil seed bank (Fenner and Thompson, 2005; Warr et al., 1993; Tessema et al., 2016) if not adjusted for the viability of said seed (Gross, 1990; Petrulaitis et al., 2022). Seed extraction is typically performed by sieving (Roberts, 1981) or flotation in oversaturated salt solutions (Malone, 1967; Tsuyuzaki, 2006).

In contrast, seedling germination is effective in determining seed viability and species but requires time, space, and suitable conditions for cultivation. In addition, not all conditions for the germination of different species are met by a single cultivation procedure (de Villiers et al., 1994). It also has a higher risk of external contamination of seed samples (Karlík and Poschlod, 2014). Some viable seeds may not germinate due to dormancy or specific environmental requirements for germination (Newton et al., 2020; Flanigan et al., 2020), and some germinating seeds may not live up to the seedling phase, leading to an underestimation of the soil seed bank (Baskin and Baskin, 2014). It is stated that up to 90% of wild plant species produce seeds that are dormant (Kildisheva et al., 2020; Klupczyńska and Pawłowski, 2021). To reduce the amount of soil used, the emergence method can be combined with the extraction method by sieving samples before germination. According to Ter Heerdt et al. (1996), this approach has germination rates varying between 81% and 100% of the viable seeds present in the soil sample.

While seedling germination is the most commonly used method (Padonou et al., 2022), the comparison of both methods is important for the correct analysis of the soil seed bank (Gross, 1990). Previous research has used both methods in a single habitat, but such studies are limited (Brown, 1992; Gross, 1990; Roberts, 1981; van Etten et al., 2014; Leon et al., 2015; Leon and Wright, 2018). For example, Brown (1992) estimated higher densities of seeds and diversity in forests using the extraction method. In tallgrass prairie, Johnson and Anderson (1986) also found higher densities using extraction, but higher diversity using cultivation. According to Reinhardt and Leon (2018), seed extraction method yields 418% higher seed density and 35% more species per sample than seedling emergence method. Different estimates of the soil seed bank using each method have led some authors to recommend the use of both seedling germination and seed extraction methods simultaneously (de Villiers et al., 1994). Price et al. (2010) recommend conducting a pilot study first to determine the detectability of individual species and then decide what method to use.

## 2 Materials and methods

**Figure 1:**
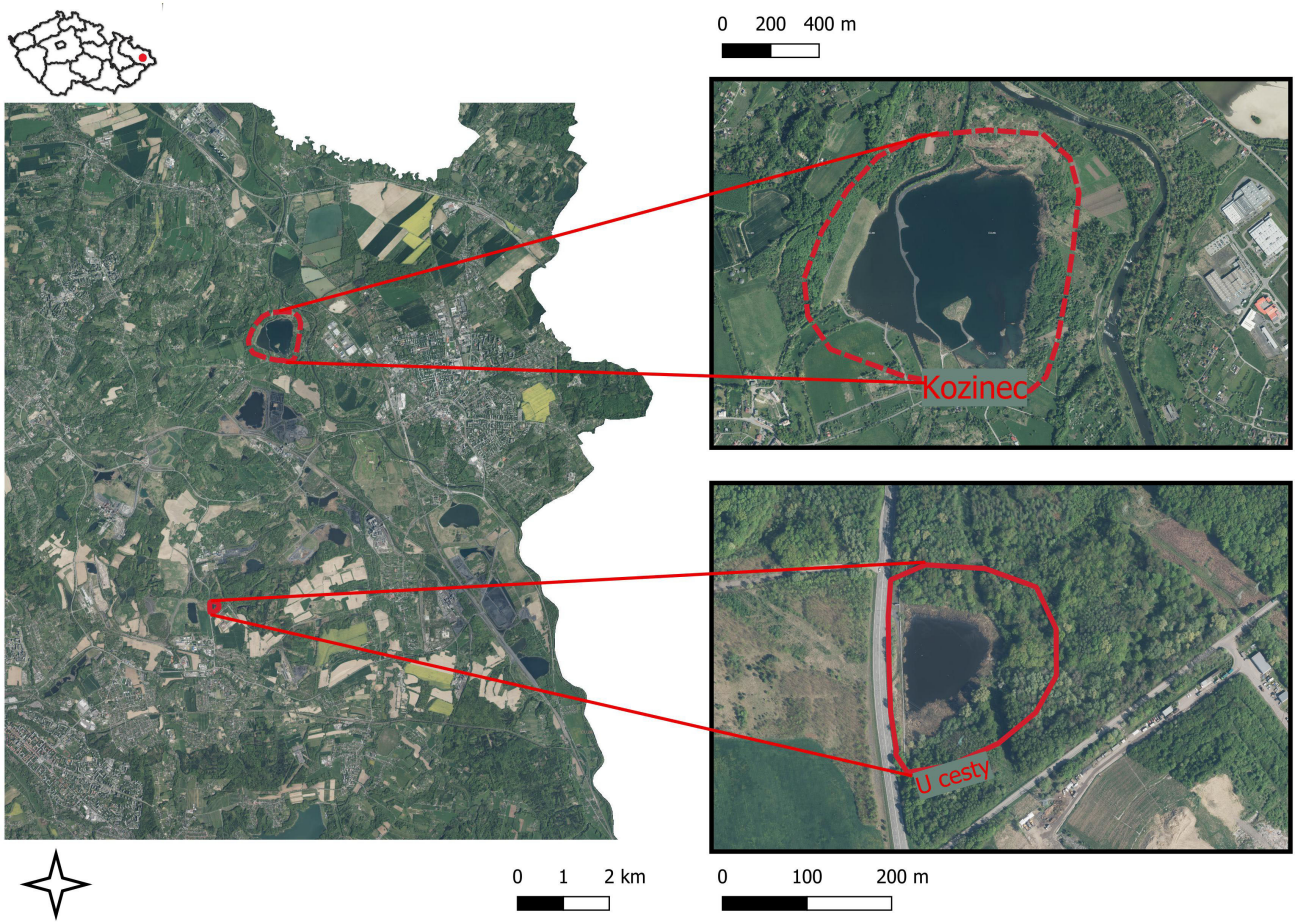
Research localization. Map created using the Free and Open Source QGIS.

The study was carried out in the subsidence basins in the Karvina region of Czech Republic, which is part of the Ostrava – Karvina District in the Upper Silesian black coal basin. For soil seed bank research, two model subsidence basins were chosen (**Figure 1**). The analysis of above-ground vegetation and soil seed bank took place in the littoral area and adjacent riparian vegetation of forested and non-forested habitats. The Kozinec subsidence basin (49°52’0.150”N, 18°29’25.752”E) is a large water body covering 70 hectares, located in the municipality of Doubrava. It is divided by human-made peninsulas into the non-flowing Kozinec Lake and the western riverine area of the Karvinský Creek. The studied habitats included littoral zones, ravine talus forests, and permanent grasslands. The substrate at the sampling sites reflects the area’s original agricultural use, consisting of light loamy to clay soils with a seed bank containing diaspores from the region’s native plant communities.

The subsidence basin U cesty (49°48’46.865”N, 18°28’40.736”E) is located in the Karviná region within the Moravian-Silesian Region of the Czech Republic (**Figure 1**). This smaller subsidence basin, covering an area of 2 hectares, lies north of the Horní Suchá municipality. The reservoir banks were rehabilitated using tailings from the Karviná mines. The tailings consist of coarse-grained, dark, sharp-edged Carboniferous rock fragments, which are entirely sterile and lack preserved plant propagules. In the western and southwestern sections of the basin, the compact tailings substrate supports scattered patches of reed growth. In the northern section, the tailings were partially covered with clay-loam soil and subsequently afforested, although larger exposed tailings areas remain visible. The biotopes in this basin predominantly include reed-dominated areas, barren wastelands with sparse vegetation, and ruderalized forests.

The following characteristics were evaluated at the model areas:

- Analysis of above-ground vegetation - botanical and phytosociological survey.
- Composition of the soil seed bank using cultivation and extraction methods.
- Determining the similarity of the soil seed bank and above-ground vegetation and the soil seed bank within the cultivation and extraction method.
- Determining the effect of forested and non-forested habitats on the quality and quantity of the soil seed bank.

### 2.1 Above-ground vegetation research

The above-ground vegetation was evaluated using phytosociological techniques of Zurich – Montpellier school (Braun-Blanquet, 1964). In each sampling area, a 100 m2 square was designated, and all plant species were identified and recorded three times a year. The phytosociological relevés were placed to include both forested and non-forested habitats. The coverage of each species was determined using the Braun-Blanquet scale. The vegetation recorded in each phytosociological relevé was then classified into phytosociological alliances. A total of 30 phytosociological relevés were evaluated - 15 for each subsidence basin.

### 2.2 Soil seed bank research

Sampling was carried out in 15 locations within each subsidence basin following the phytosociological relevés. Samples for extraction method were taken in May of 2019 for spring aspect; in November 2019 for autumn aspect. For cultivation method in May 2020. Three areas in the littoral of Kozinec were combined into one after a spring seed extraction analysis. At each location, five soil cores were randomly taken from a 2 m radius circle using a 5.3 cm diameter and depth cylinder. The depth was chosen with knowledge of the predominant occurrence of seeds in the upper layers of the soil (Leck and Simpson, 1987; Warr et al., 1994). These five soil cores were pooled into a single mixed sample. However, autumn samples from 5 areas were not taken for cultivation analysis due to flooding of the subsidence basin U cesty. During the reconnaissance of the territory in the following years, it was found that this is a permanent flood and the expansion of the water body which is probably caused by ongoing relief subsidence. In total, 540 soil cores were collected, forming 108 mixed samples.

The mixed soil samples were transported to the laboratory for analysis. The samples were processed by washing with a 0.2 mm sieve. The samples for extraction method were then dried and the seeds were manually extracted, counted, and identified using binocular magnifier CarlZeiss Stemi DV4 8x-32x (especially for small seeds). The samples for cultivation analysis were washed using a sieve with the same mesh size. Each mixed sample was then spread thinly on the surface of a substrate suitable for seed germination and seedling establishment in 20-cm diameter pots. The samples were watered daily, and the germinated seedlings were identified, counted, and removed from the pot.

The average seed length (mm) and the weight of 1000 seeds (g) of the captured plant species in the soil seed bank were determined. For this purpose, tabular values from the online databases The Digital Plant Atlas (2006) (seed length) and The Seed Information Database (2023) (average weight of 1000 seeds) were used.

### 2.3 Statistical analysis

The collected data was analyzed using a one-way analysis of variance (ANOVA) or Kruskal-Wallis test according to their distribution (determined by Shapiro – Wilk test) with α = 0.05. To gain further insight, the species identified in the soil seed bank were grouped according to Raunkiaer’s life strategy and dispersal strategy (Sádlo et al., 2014). The high variance in the number of seeds extracted and the seed mass of different species led to a log transformation (ln+1) to better visualize the results, which were then processed and represented graphically. The similarity between the above-ground vegetation, the cultivation method, and the extraction method of the soil seed bank analysis was calculated using the qualitative Sørensen similarity index due to a different calculation of species abundance (percentage intervals for above-ground vegetation and total numbers in the soil seed bank). To compare the similarity of the results of cultivation and extraction methods, Non - Metric Multidimensional Scaling (NMDS) analysis was chosen for quantitative data and supplemented with the Sørensen index.

The estimate of the number of species (sensu lato biodiversity) was calculated with respect to the ratio of sample sizes using rarefaction. Rarefaction curves were compiled from a series of samples for each method. Rarefaction curve for a combination of both methods was formed using combined series of samples where each sample determined by the extraction method was followed by a sample of the same location determined by the cultivation method (e.g. 1. extraction, 1. cultivation, 2. extraction, 2. cultivation) The construction of the curves was based on a number of unique species determined by the cultivation and extraction method, where unique species means the 1st record of the given species in the data file. Logarithmic trendlines with their equations and the coefficient of determination (R²) were established.

Statistical analysis and visualization of the data was carried out in R software (R Core Team, 2021) with Vegan package (Oksanen et al., 2020) and MS Office.

## 3 Results

### 3.1 Above-ground vegetation

In the above-ground vegetation, 115 plant species from 43 families were identified, with a predominance of the *Asteraceae, Poaceae*, and *Rosaceae* families. Plant communities mainly consist of initial vegetation communities on undeveloped soils (phytosociological alliances *Dauco carotae-Melilotion* and *Fragarion vesceae)*, mesophilic ruderal and semi-natural vegetation (*Aegopodion podagrariae)*, that transition into mesophilic meadows (*Arrhenatherion elatioris*), Reed communities *(Phragmition australis)* appear on the banks of waterlogged basins and alder stands (*Alnion incanae*) develop in periodically flooded habitats. Secondary oak forests (*Quercion roboris*) also appear in humid habitats.

### 3.2 Soil seed bank

**Figure 2:**
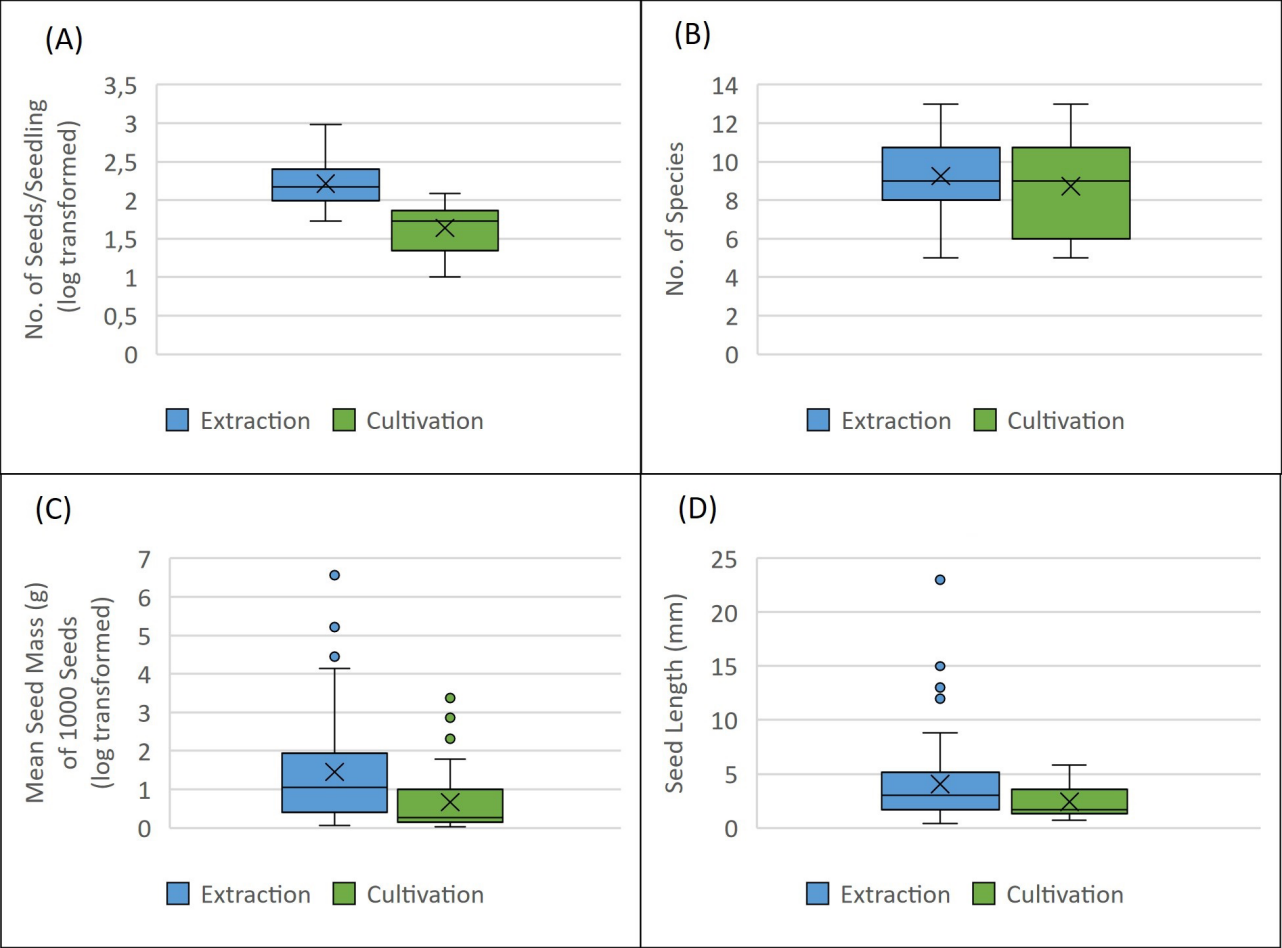
**(A)** Number of seeds or seedling identified in extraction and cultivation method (y axis is log transformed); **(B)** Number of unique species identified in each method; **(C)** Seed mass of species identified in each method (g per 1000 seeds; y axis is log transformed); **(D)** Seed length (mm) if identified species.

Using the cultivation method, 60 plant species from 24 families were identified, with a pronounced prevalence of Asteraceae and Poaceae, based on 1,580 seedlings. In contrast, the extraction method yielded a total of 5,999 seeds from 66 plant species across 25 families, again predominantly from Asteraceae and Poaceae families. In particular, 53.11 ± 31.07 seedlings were observed on average per sample in cultivation analysis, while extraction analysis yielded an average of 203 ± 181 seeds (**Figure 2A**).

The most numerous species in the case of both methods was nitrophilous ruderal *Urtica dioica*, which accounted for 54% of all determined seedlings and 47% of all captured seeds. Germinated seedlings and separated seeds of other species were more sporadic in the soil; among the relatively more numerous, we can mention the seedlings of *Poa* spp. (13%); representatives of thermophilic ruderal vegetation *Chenopodium album* (6%), *Hypericum perforatum* and *Glechoma hederacea* (3%); species of mesophilic meadows *Festuca rubra* (2%) and seeds of pioneer tree species *Betula pendula* and *Alnus glutinosa* (13%); species of glades and bright disturbed habitats *Rubus idaeus* (9%); and species associated with floodplain forests *Salix alba* (5%); *Carex riparia* (3%) and *Sambucus nigra* (2%). It can therefore be said that a very similar number of species is captured using the cultivation method as well as the extraction method (60 and 66). The fundamental difference is in the number of captured individuals (seedlings/seeds).

The effect of the tree layer of the stand (“forest habitats vs. “non-forest habitats”) on the abundance of the soil seed bank was not statistically significant for the cultivation or extraction method (ANOVA; p = 0.204). Specifically, in the cultivation of samples from forest habitats, an average of 53.06 ± 32.19 seedlings was observed, compared to 53.2 ± 30.63 seedlings in forestless habitats. The extraction method resulted in an average of 207.67 ± 127.28 seeds in forest habitats and 196.75 ± 247.61 seeds in forestless habitats. The effect of the presence of the tree layer was statistically significant in the case of the number of species in the soil seed bank using the extraction method (ANOVA; p = 0.016), in the case of the cultivation method it was not statistically significant (ANOVA; p = 0.276). Soil seed bank analysis revealed an average of 8.71 ± 2.39 plant species when using cultivation analysis and 9.2 ± 2.01 species with extraction analysis (**Figure 2B**).

In terms of life strategy, most of the species found in the soil seed bank belong to hemicryptophytes with a dispersal strategy Allium and Epilobium. These species are characteristic of dry and mesic habitats and primarily disperse via autochory or anemochory. Phanerophytes were more frequently represented in the extraction method, only *Alnus glutinosa*, *Rubus idaeus* and *Robinia pseudoacacia* appeared in the cultivation method (**Table 1**).

**Table 1:** Common species in both analysis methods.

*Species*	*Life form*	*Dispersal strategy*	*Seeds in extraction*	*Seedlings in cultivation*
*Alnus glutinosa*	Phanerophyte	*Epilobium*	760	24
*Anthoxanthum odoratum*	Hemicryptophyte	*Allium*	1	1
*Artemisia vulgaris*	Hemicryptophyte	*Allium*	20	3
*Bellis perrenis*	Hemicryptophyte	*Allium*	1	3
*Calamagrostis epigejos*	Hemicryptophyte	*Epilobium*	21	8
Carex sp.*	Hemicryptophyte	*Allium*	170	18
*Cirsium arvense*	Cryptophyte	*Epilobium*	3	5
*Conyza canadensis*	Therophyte	*Epilobium*	1	1
*Daucus carota*	Hemicryptophyte	*Bidens*	8	2
*Galium aparine*	Therophyte	*Bidens*	2	8
*Hieracium sp.*	Hemicryptophyte	*Epilobium*	1	7
*Chenopodium album*	Therophyte	*Allium*	10	85
*Persicaria hydropiper*	Therophyte	*Sparganium*	5	1
*Picris hieracioides*	Hemicryptophyte	*Epilobium*	31	10
Poa sp.*	Hemicryptophyte	*Allium*	63	186
Ranunculus sp.*	Hemicryptophyte	*Allium*	6	2
*Robinia pseudoacacia*	Phanerophyte	*Allium*	73	1
*Rubus idaeus*	Phanerophyte	*Cornus*	538	24
*Solidago canadensis*	Hemicryptophyte	*Epilobium*	5	8
*Symphytum officinale*	Hemicryptophyte	*Allium*	8	3
*Tanacetum vulgare*	Hemicryptophyte	*Allium*	3	10
*Taraxacum officinale*	Hemicryptophyte	*Epilobium*	4	12
*Urtica dioica*	Hemicryptophyte	*Allium*	2831	857

* means classified at the genus level.

**Figure 3:**
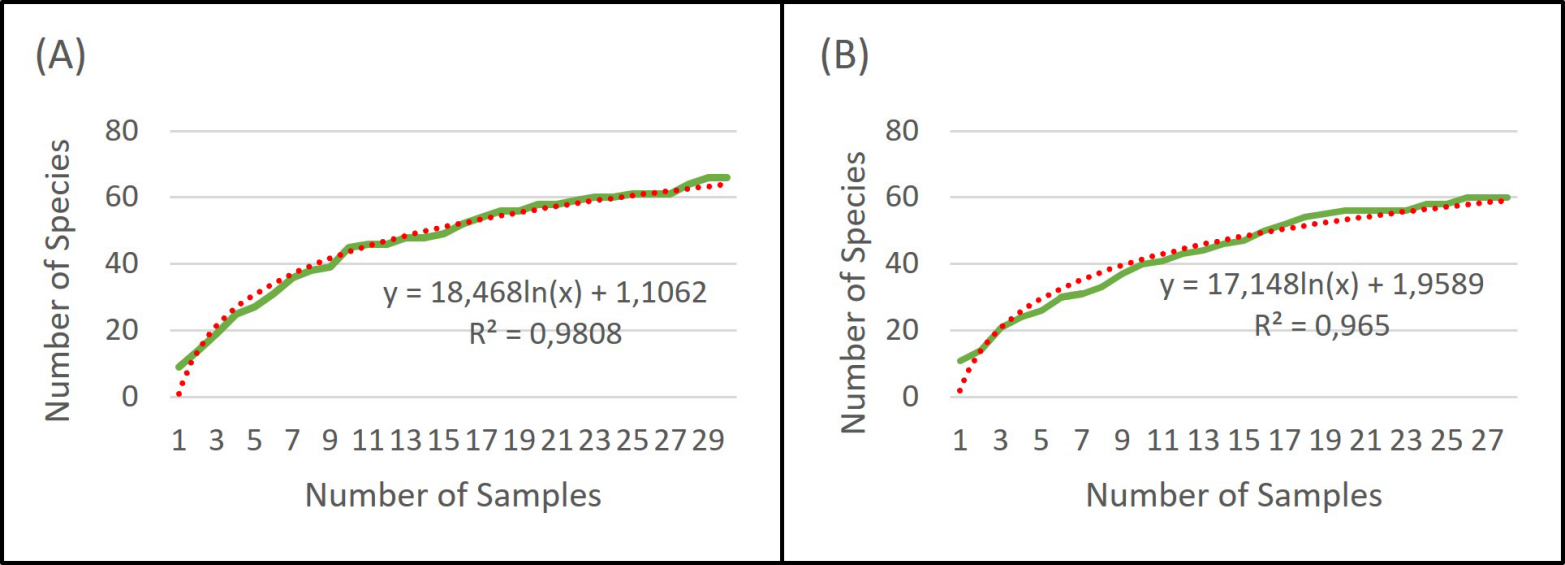
Rarefaction of extraction method **(A)** and cultivation method **(B)**.

**Figure 4:**
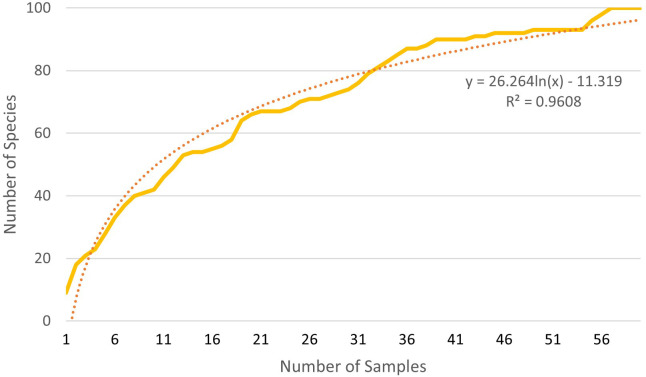
Rarefaction of both methods combined.

**Figure 5:**
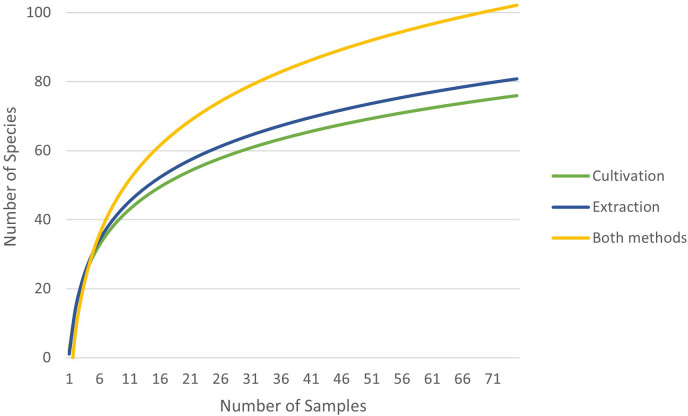
Comparison of extraction and cultivation analysis rarefaction curves and their combination.

Based on the rarefaction curve, it is evident that the number of samples we collected is sufficient for assessing the species composition of the soil seed bank. Even with a doubling of the sample size, our original number of samples would capture 86% (extraction), 84% (cultivation), and 87% (combined approach) of the plant species seeds present in the soil seed bank (**Figures 3**–**5**).

The average seed length [mm] as characterization of seed size and seed mass of a thousand seeds [g] was established for the seed of each captured species. Analysis revealed that the method used had statistical significance for both length (Kruskal – Wallis; p = 0.0012) and mass (Kruskal – Wallis; p = 0.00047) of seeds captured in the soil seed bank with α = 0.05. The average seed mass of 1000 seeds was 24.398 ± 11.419 g in extraction and 2.16 ± 0.644 g in cultivation, as seen in **Figure 2C**. The average length of seeds captured by extraction method was 4.064 ± 0.454 mm and average length of seeds of seedling established by cultivation method was 2.401 ± 0.188 mm (**Figure 2D**).

### 3.2 Similarity of soil seed bank and above-ground vegetation

The similarity between the aboveground vegetation and the soil seed bank determined by the extraction method was 41,62% and the similarity between the aboveground vegetation and the cultivation method was 40%. The Sørensen similarity index between the cultivation and separation methods was only 38.33%. Combining both soil seed bank analysis methods, the similarity between the soil seed bank and the above-ground vegetation reached a higher value of 51.46%. Since the extraction method tends to record higher numbers of woody species, we also calculated similarities between above-ground vegetation and soil seed bank only for forest and non-forest areas. In non-forest areas, the Sörensen similarity index reached 36.036% between above-ground vegetation and cultivation analysis and 38.261% between above-ground vegetation and extraction method, with a similarity of 33.333% between the two soil seed bank analysis methods. In forest biotopes, similarity reached values of 39.37% between above-ground vegetation and cultivation method; 26.087% between above-ground and separation method, and 24.176% between extraction and cultivation analysis.

**Figure 6:**
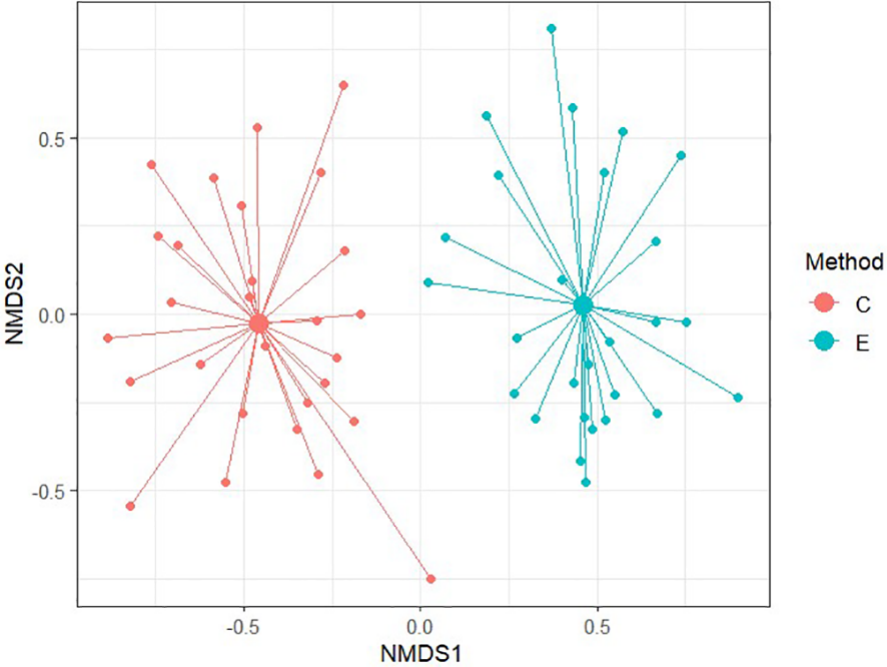
Diagram of Non-metric multidimensional scaling between chosen methods for assesing their similarity. Stress value = 0.225.

**Figure 7:**
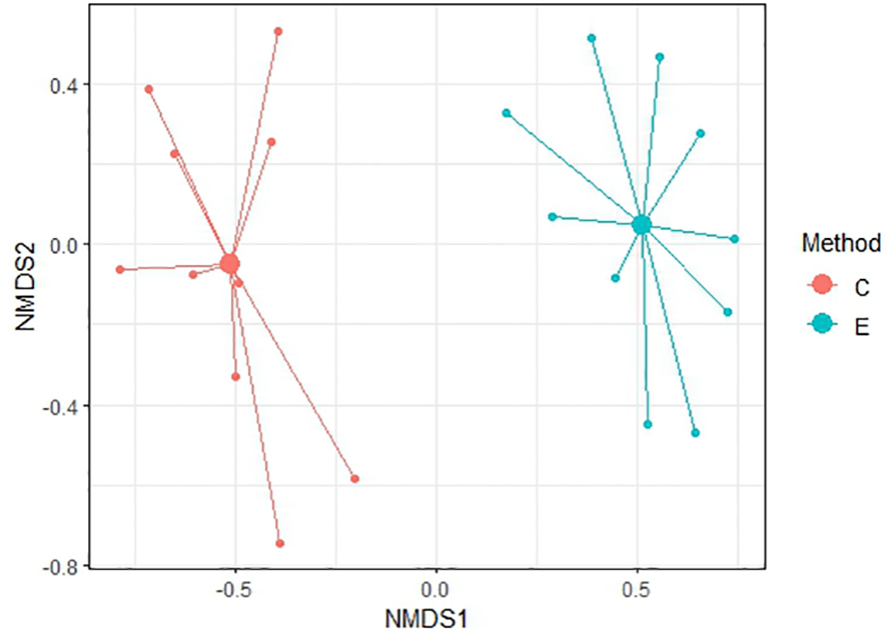
NMDS analysis between two methods in forest habitats. Stress value = 0.184.

**Figure 8:**
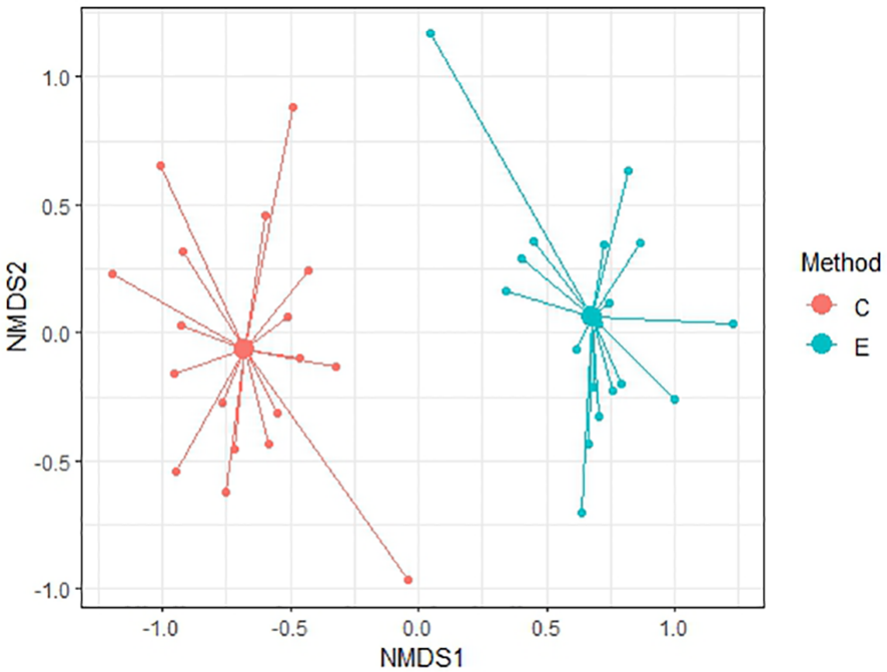
NMDS analysis between two methods in non-forest habitats. Stress value = 0.174.

To compare similarity between each method of soil seed bank research, Non-metric Multidimensional Scaling (NMDS) was conducted. NMDS was performed to two dimension using Bray-Curtis distance for comparison of relevés of 56 samples and subsequently only for 20 non-forest samples and 36 forest samples. Ordination provided better results in comparison of non-forest and forest samples with stress values reaching 0.174 and 0.184, respectively. For all 56 samples, NMDS analysis is harder to interpret as the stress level is 0.225 which can imply a greater informational loss by the two-dimensional representation in a higher number of samples. According to **Figures 6**–**8** it is clear that there are substantial differences among seed compositions of samples established by cultivation and extraction analysis.

**Figure 9:**
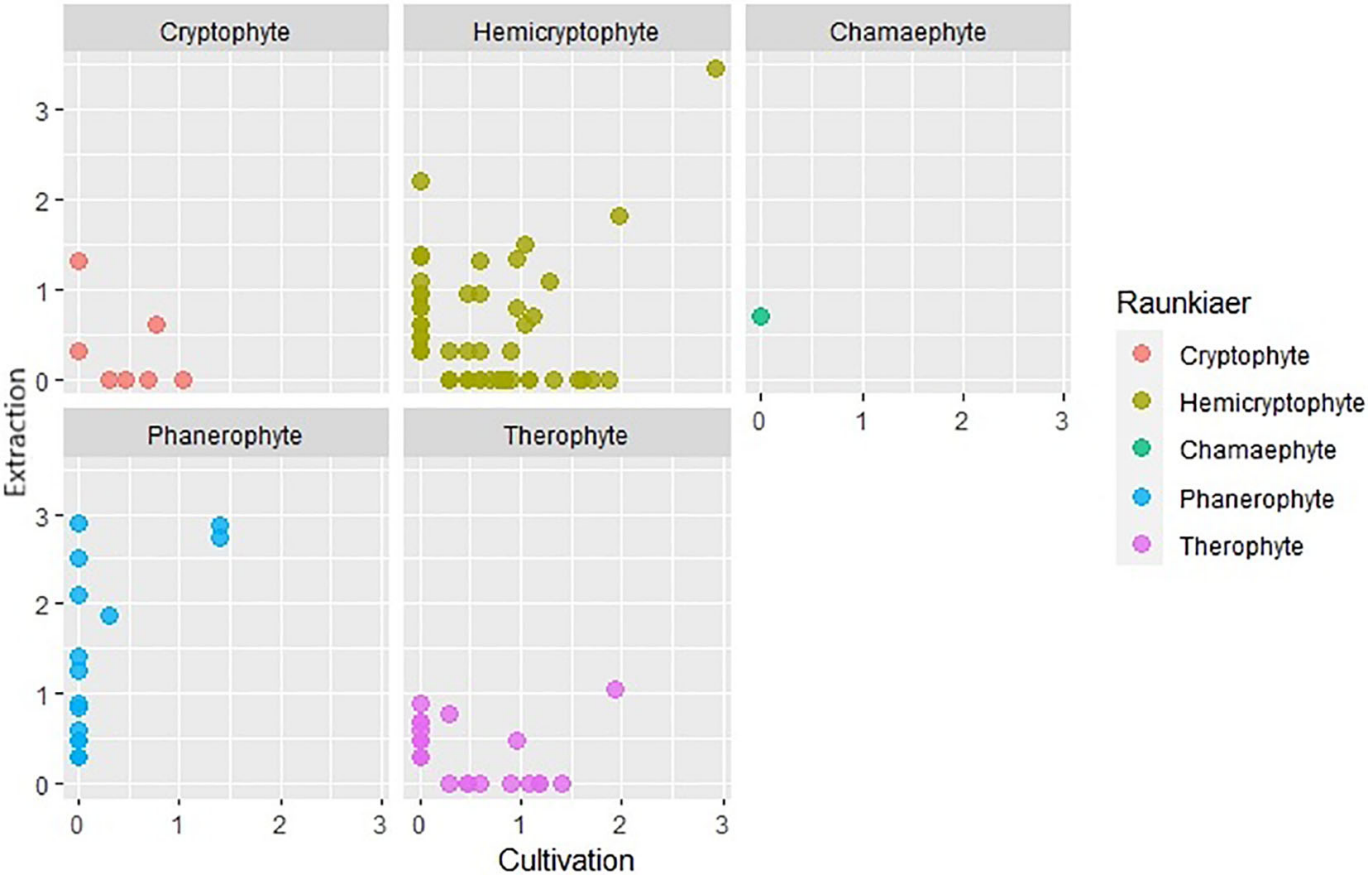
Comparison of number of species captured by extraction and cultivation method based on Raunkiær plant life-form.

**Figure 10:**
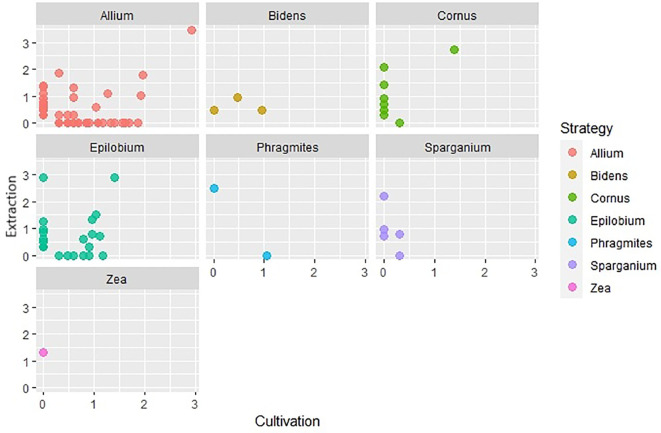
Comparison of number of species captured by extraction and cultivation method based on their dispersal strategy.

Using different soil seed bank analysis methods, plants characterized by distinct life forms and specialized dispersal strategies often show varying levels of success of detectability. We created scatter graphs from logarithmically transformed (log+1) data of the number of seeds and seedlings captured for each life form a dispersal strategy. The x-axis in these graphs represents the number of established seedlings in cultivation analysis and the y-axis represents the number of seeds captured in extraction analysis. Based on these plots (**Figures 9**, **10**), it was observed that cryptophytes, therophytes, and hemicryptophytes were effectively detected by both the extraction and cultivation methods. The extraction method, however, proved particularly suitable for identifying phanerophytes and chamaephytes, offering advantages in detecting species with dispersal strategies similar to Cornus (species spreading by autochory and endozoochory, typical of herbs and smaller woody plants with fleshy fruits) and Zea (species dependent on humans for reproduction, nearly always non-native, including agricultural and horticultural cultivars). In contrast, both methods were equally effective at identifying species with dispersal strategies like those of Allium and Epilobium.However, it is important to note that a significant number of plant species were exclusively detected by one of the two analysis methods, highlighting the nuanced intricacies of soil seed bank analysis for different species and life forms.

## 4 Discussion

### 4.1 Composition of soil seed bank

A total of 7579 seeds were identified. Cultivation recorded fewer seeds than extraction (20.8% and 79.2%, respectively). This corresponds to other studies that compared these two methods (Brown, 1992; Chiquoine and Abella, 2018; Gandía et al., 2022). For instance, de Villiers et al. (1994) found 4-10 times more seeds using extraction compared to cultivation. The number of germinated seeds is mainly influenced by their dormancy, the ability to break this dormancy, and successfully establish seedlings (Bernhardt et al., 2007; Brown, 1992). Using the combined method of rinsing through a sieve, we can expect a positive effect of seed scarification, which can open seeds with a hard coat or testa. Light damage to the coat allows water and gases to penetrate the seed, leading to breakage of the dormancy. This positive effect has been demonstrated, for example, in the seeds of *Robinia pseudoacacia* and *Rubus idaeus*, identified using the cultivation method of soil seed bank analysis (Contreras et al., 2016; Wada and Reed, 2011). The time between sampling, processing, and planting also has an impact. For example, willow seeds are short-lived, and the viability of their seeds diminishes within a few days at room temperature (Maroder et al., 2000), which could be a reason for the unsuccessful germination of these seeds, although we found a significant number of seeds of this species in the soil using the extraction method. On the other hand, seeds of some species require pretreatment to germinate (Kozlowski and Pallardy, 2002).

Regarding plant species, *Urtica dioica* dominated the soil seed bank of the subsidence basins. The low mass of *Urtica dioica* enables the production of large numbers of persistent seeds (Thompson and Grime, 1979) and few of the seeds germinate in the period immediately following dispersal, leading to a large seed bank that changes little in size with season (Taylor, 2009) so the species frequently dominates the soil seed bank as found in many studies (Eycott et al., 2006; Gioria and Osborne, 2010; Jankowska-Błaszczuk et al., 1998). Another abundant species in the soil seed bank, *Chenopodium album*, also frequently dominates in highly disturbed biotopes (Janicka, 2006; Kim and Lee, 2005; Schnee et al., 2023; Skuodienė and Matyžiūtė, 2022). In the extraction method, the pioneer trees *Betula pendula* and *Alnus glutinosa*, which produce large amounts of seeds (Claessens et al., 2010; Perala and Alm, 1990; Van Splunder et al., 1995), are abundant. On the contrary, the grasses and weeds were more successful in cultivation analysis.

The germination of phanerophyte seeds can also be impacted by their quality, which corresponds to the condition of the maternal plant arising from the habitat conditions. In habitats impacted by anthropogenic activities with higher concentrations of hazardous elements, there can be a reduction in pollen and seed quality, resulting in decreased seed germination (Franiel and Kompała-Bąba, 2021). Subsidence basins of interest are characterized by increased concentrations of chromium and arsenic (Plohák et al., 2022), which have been shown to harm plant generative parts (Liu et al., 2005; Speranza et al., 2007; Zeid, 2001).

Using rarefaction, we can verify whether combining cultivation and extraction analysis leads to an increase in the number of captured species in the soil seed bank or if it is simply a result of increasing sample numbers. In 57 samples, consisting of 30 samples determined by the extraction method and 27 samples determined by the cultivation method, 82 unique plant species were identified. Using only the extraction method, according to the trend line, 57 samples would capture 76 unique plant species, and the cultivation method would capture 71 unique plant species, conversely.

### 4.2 Seed characteristics

It is commonly stated that extraction analysis is inappropriate for small seeds that can be lost during sowing (Brown, 1992; Mesgaran et al., 2007). In our research, even samples for cultivation analysis were sown through sieves, but it is evident that the extraction method still has a bias towards longer, wider, and bulkier seeds – the mean value of the seed length was 4.06 mm with a median of 3.05 and the mean mass of 1000 seeds was 24.397 g with a median of 1.88 g. In cultivation method, the mean value of seed length was 2.41 mm with a median of 1.7 and the mean seed mass of 1000 seeds was 2.16 g with a median of 0.31. In extraction analysis, this mainly concerns seeds of woody species such as *Prunus domestica*, *Fraxinus excelsior*, *Cerasus avium*, *Cornus max*, and *Citrullus lanathus* (residue of human activity). In the cultivation method, the largest and most massive seeds belong to perennial herbs such as *Daucus carota*, *Beta vulgaris*, *Symphytum officinale*, *Calystegia sepium*, and one of the few germinated seeds of woody species, *Robinia pseudoacacia*. These results are consistent with de Villiers et al. (1994), who found that the extraction method captures seeds of shrubs and trees in addition to small-seeded species. Gonzalez and Ghermandi (2012) achieved a large seed recovery of seeds heavier than 0.3 mg and longer than 1 mm in grasslands using extraction analysis, while this method was not suitable for small-seeded species. According to Price et al. (2010), extraction results in the loss of seeds smaller than 2 mm, Similarly, Mesgaran et al. (2007) found a decrease in the effectiveness of the extraction method for seeds smaller than 1 mm.

### 4.3 Similarity of soil seed bank and above-ground vegetation

A higher similarity between the soil seed bank and above-ground vegetation is primarily observed in unstable habitats with a regular disturbance régime, to which plant species have adapted by forming long-lived seeds (Grime, 2002; Matus et al., 2005). In stable habitats, the possibility of seed germination is low due to a lack of open spaces. In these habitats, more plants produce short-lived seeds or reproduce vegetatively (Bekker et al., 1997; Lee, 2004; Matus et al., 2005). In the subsidence basins studied, there is stable forest vegetation but also unstable vegetation of disturbed habitats.

The similarity results between each method and the above-ground vegetation were similar in the subsidence basins. The similarity reached 40.476% between cultivation and above-ground vegetation and 40.909% between extraction and above-ground vegetation. These values are within the usual range of soil seed bank and above-ground similarity (Johnson and Anderson, 1986). They also correspond to similarities observed in many degraded habitats, such as degraded forests in the temperate zone (Kwiatkowska-Falińska et al., 2011); degraded high-mountain meadows (Shang et al., 2016); and former arable fields (Dölle and Schmidt, 2009).

We observed a very low similarity between the determination of the soil seed bank by cultivation and extraction methods using NMDS analysis and Sørensen similarity index (37.4%). The soil seed bank is temporally and spatially highly variable, and a large portion of seeds of many species are dispersed only near the maternal plant (Clark et al., 2005; Schupp and Fuentes, 1995). However, in our study, soil samples were taken from the same location within a radius of 2 m, so spatial heterogeneity should be reduced.

A notable difference between the two methods is apparent in terms of the life forms of plants. In most life forms, unique species were captured by both extraction and cultivation analysis. Chamaephytes, for which only one plant species was recorded and phanaerophytes were exceptions. No phanerophyte was determined by cultivation analysis alone, while a large portion of species was determined only by extraction analysis. Physical and physiological dormancy occurs in many species of phanerophytes (Wada and Reed, 2011). Physical dormancy is caused by an impermeable seed coat or testa that functions as a mechanical barrier that prevents the emergence of seedlings that must be weakened to allow germination (Baskin and Baskin, 2014). Mechanical or chemical scarification of seeds promotes the germination of seeds with physical dormancy unless there is another factor, such as physiological dormancy, stopping the germination process (Baskin and Baskin, 2014). Seed embryos of some species lack maturity even after they are shed from their parent trees and require a few weeks of stratification at specific temperatures to mature (Baskin and Baskin, 2014). For these reasons, there is a small chance that most phanerophytes can be grown from the soil seed bank without special treatment. In our study, there were a few species of phanerophytes with dormant seeds that germinated. The seeds of *Rubus idaeus* exert both physical and physiological dormancy commonly broken by cold stratification or/and the use of sulfuric acid (Hall et al., 2009). The seeds of *Robinia pseudoacacia* have physical dormancy and need to be scarified to increase germination (Masaka and Yamada, 2009). It is therefore possible that their ability to germinate was enhanced by scarification, which may have occurred during sieving prior cultivation. However, there are many species of phanerophytes whose seeds were found only by the extraction method. Many of these seeds are in physical, physiological, and morphological dormancy or in their combination. The seeds of *Carpinus betulus and Cerasus avium* need warm-cold stratification; on the other hand, the seeds of *Fraxinus excelsior* need cold-warm stratification (Eşen et al., 2006; Güney et al., 2015; Raquininsta et al., 2002). The germination of *Alnus glutinosa* seeds is enhanced by a short period of moist chilling, and *Cornus* sp. *seeds* require a longer period of cold stratification (Gosling et al., 2009; Kollmann and Grubb, 2001). Of course, other cues can be used to break seed dormancy (Bungard et al., 1997). *Sambucus nigra* seeds need scarification in addition to stratification (Leif et al., 2011). On the other hand, some seeds are not dormant and do not require any pretreatment (*Ulmus glabra*) or seeds that lose viability quickly after shedding from the parent tree, so prolonging seed planting is contra-productive (Cicek and Tilki, 2006; Maroder et al., 2000).

Even with successful cultivation, the number of seedlings for some phanerophyte species reached only a fraction of the seeds found during extraction - *Alnus glutinosa* (24/760); *Robinia pseudoacacia* (1/73); *Rubus idaeus* (24/538).

## 5 Conclusion

The soil seed bank, along with its function and dynamics, plays a crucial role in the process of succession, yet it has been relatively overlooked in areas requiring restoration. However, understanding the soil seed bank and its relationship to above-ground vegetation is essential for preventing failures in reclamation efforts and for predicting the potential development of plant communities. In waterlogged subsidence basins - important areas of post - hard coal mining landscape, the method chosen for soil seed bank analysis affects the number, length, and weight of captured seeds. The number of recorded plant species, however, is not significantly impacted by the method. The extraction method captures four times more seeds than seedling cultivation, and the seeds collected through extraction are longer and heavier. Although both methods result in similar soil seed bank and above-ground vegetation similarities, each method captures a significant number of different species. The difference is particularly noticeable in phanerophytes with the Cornus dispersal strategy. For a comprehensive analysis of the soil seed bank, we recommend combining seed extraction with cultivation, which results in more species captured and higher similarity between soil seed bank and above-ground vegetation. However, number of samples must be increased accordingly and since this is a time-consuming and labor-intensive process, it is advisable to first assess the suitability of each method for the study’s objectives, habitats, and soil conditions in the target areas and choose accordingly.

## Data Availability

The raw data supporting the conclusions of this article will be made available by the authors, without undue reservation.
